# Zero Shear Viscosity of Hybrid Modified Asphalts and Its Gray Correlation with Other Properties

**DOI:** 10.3390/ma15207056

**Published:** 2022-10-11

**Authors:** Wei Wang, Baomin Chen, Junan Shen

**Affiliations:** 1 School of Civil Engineering, Chongqing Jiaotong University, Chongqing 400074, China; 2 Jiangsu Provincial Center of Ecological Road Technology Industrialization and Research, Suzhou University of Science and Technology, Suzhou 215011, China; 3 Department of Civil Engineering and Construction, Georgia Southern University, Statesboro, GA 30458, USA

**Keywords:** SBS, crumb rubber modifier, hybrid modified asphalt, zero shear viscosity

## Abstract

The viscosity of modified asphalt binders is the most important property to ensure the durability of open-graded friction course (OGFC). Zero shear viscosity (ZSV) is considered to be the optimum result to reflect the rutting characterization of high viscosity modified asphalt binders, compared with conventional vacuum capillary viscosity. However, there are few reports on using ZSV to evaluate the material characteristics of hybrid modified asphalt binders and to establish the relationship between ZSV and other properties. In this paper, a high viscosity hybrid modified asphalt binder was prepared with Sty-rene-butadiene-styrene (SBS) and Crumb rubber modifier (CRM). ZSV, three major indicators, 60 °C dynamic viscosity, 135 °C Brookfield viscosity, and a dynamic rheological test were used to determine the properties of the hybrid modified asphalt binders. The relationship between ZSV and other properties was studied by the gray correlation analysis method. Results indicated that: (1) The viscosity of hybrid modified asphalt binders increases with the decreasing frequency. When the frequency tends to 0, the viscosity of asphalt at this time is zero shear viscosity; (2) The values of the ZSV of hybrid modified asphalt binders have a large increase as the dose of both CRM and SBS modifiers were increased; and (3) The ZSV at 60 °C correlated well with the performance properties of rutting factor (G*/sin(θ)), indicating that the ZSV of hybrid modified asphalt binders could be a good indicator of performance.

## 1. Introduction

With the rapid development of the economy, traffic volume and heavy vehicles on pavements have gradually increased and the requirements for the strength and durability of asphalt pavements have been progressively enhanced. Consequently, the required quality of asphalt binders remains steadily high [1]. The addition of a modifier in a base asphalt binder can effectively improve the performance of the modified asphalt binder. At present, SBS and CRM are the most commonly used modifiers for this purpose. SBS and CRM mixed into the base asphalt binder mainly cause physical reactions in the modified asphalt binder. CRM are suspended in the base asphalt binder in the state of particles, absorbing part of the light components of oil in the base asphalt binder. The surface of CRM expanded to form a stable structure, and the surface of the CRM particles forms a smooth gel film increasing the coupling area with the base asphalt binder. SBS increases the mass fraction of the colloid in the asphalt binder and creates an interaction force between the SBS molecule and the asphalt molecule, which causes the asphalt binder to change from a colloidal structure to a gel structure [1]. Several studies have found that the modified asphalt binder with a single addition of CRM has poor stability and segregates the rubber particles from the base asphalt binder easily. Hybrid modification of SBS and CRM can reduce the problem of segregation and increase the viscosity of the modified asphalt binder. Economically, the hybrid modified binder can save some costs. Therefore, SBS and CRM hybrid modification of asphalt binder has gradually become popular.

A porous asphalt mixture such as Open Graded Friction Course (OGFC) and drainage asphalt mixture are usually produced using a high viscosity asphalt binder modified with SBS/CRM to prolong its durability. Asphalt binder is traditionally graded by three indexes of penetration, soften point, and ductility, and recently by performance-based grade. Viscosities at different temperatures are used as another grade index. It must be noted that any grading index should be a good indicator of the properties of asphalt binder and asphalt mixtures. The high viscosity value of modified asphalt binders is a key performance index to ensure that OGFC and drainage asphalt mixtures have sufficient strength and durability, and is usually required to be greater than 20,000 Pa·S [2]. Dynamic viscosity (absolute viscosity) at 60 °C, tested by a vacuum capillary viscometer is widely used to evaluate high viscosity modified asphalt binders. It measures the time it takes for a fixed volume of asphalt binder to flow through a capillary viscosity under closely controlled conditions of head and temperature [3]. Due to the same vacuum degree when measuring different asphalts by vacuum capillary method, the larger the viscosity of asphalts, the lower the shear rate. In fact, for non-Newtonian fluids, the viscosity is not a constant coefficient because it becomes a function of the shear rate. Therefore, the viscosity of modified asphalt binder measured by vacuum capillary viscometer is greater than the actual viscosity. This leads to a misjudgment of asphalt mixture’s resistance to permanent deformation [4].

In recent years, zero shear viscosity (ZSV) measured with a dynamic shear rheometer (DSR) has received substantial attention for this purpose [5,6]. At a vanishing shear rate/loading frequency, the pseudoplastic liquid (asphalt binder) behaves as the Newtonian liquid with a defined viscosity. This viscosity is called the ZSV [7]. Previous studies have shown that ZSV value has a good correlation with rutting performance of asphalt mixtures [8]. At present, ZSV is generally calculated by long-term creep tests and oscillatory tests. Creep tests require hours or even days, and it is difficult to determine when steady-state flow is obtained. The oscillation test is a more common method, which models ZSV using complex viscosity corresponding to vanishing frequency [6,9]. Different models can be adopted to extrapolate the ZSV, such as the Cross model, Carreau model, Carreau–Yasuda model, etc. [10].

Nowadays, ZSV has been widely used to evaluate the influence of asphalt modifiers on the high-temperature properties of asphalt binders, and it is considered as a good substitute for the vacuum capillary viscosity [11,12,13]. Geng and Li [14] tested the ZSV and dynamic viscosity of 60 °C for 12 kinds of modified asphalt binders with high viscosity and found that dynamic viscosity at 60 °C was prone to a virtual high and the ZSV could reasonably characterize the bonding characteristics of asphalt binders in pavement structure. The idea of using ZSV as a key index for viscosity evaluation of high viscosity modified asphalt binder was then proposed. Li, Geng, and Sun [15] used the dynamic shear rate scanning method to obtain the capillary viscosity and ZSV of asphalt binder, analyzing the shear rate level of asphalt binder with two kinds of viscosity and the shear rate level of asphalt binder on pavement structure, which indicated that the shear rate of the first Newtonian flow zone corresponding to the ZSV is consistent with the shear rate of the asphalt binder in the pavement structures of the ZSV, and it can be more reasonable than the dynamic viscosity to characterize the bonding characteristics of high viscosity modified asphalt binder, and better reflect the bonding effect of OGFC mixtures according to the scattering test results. Wang [16] used the relationship between the viscosity and shear rate at low frequency to obtain their ZSV for base asphalt binder. Since modified asphalt binder cannot reach the viscous flow state at a low frequency, the ZSV can only be obtained by a rheological model or a static single cycle creep recovery test. Fan and Song [17] selected the two flow models of Carreau and Cross, and they used lingo and origin calculation software to simulate the asphalt binder ZSV, indicating that the Carreau model can better simulate the ZSV of the modified asphalt binders than the Cross model. A gray correlation analysis between zero shear viscosity and rheological properties shows that the irreversible creep compliance, rutting factor, and ZSV of asphalt binder can better evaluate the performance of asphalt binder at a high temperature. Therefore, the gray correlation analysis proves that ZSV characterizes the high temperature performance of asphalt binder more reasonably [18].

In summary, ZSV can better characterize the high temperature performance of high-viscosity modified asphalt binders and can also prevent the measured viscosity of the modified asphalt binder being falsely high. However, most of the research results use a single modified asphalt and focus on the relationship between ZSV and dynamic viscosity. There are only a few reports on using ZSV to evaluate the material characteristics of SBS/CRM hybrid modified asphalt binders, and there is a lack of research on the relationship between ZSV and other properties (three major indicators, among others) of hybrid modified asphalt binders. In this paper, ZSV, three major indicators, 60 °C dynamic viscosity, 135 °C Brookfield viscosity, and a dynamic rheological test were used to determine the properties of the hybrid modified asphalt binders. The relationship between ZSV and other properties was studied by the gray correlation analysis method. Thereby providing an alternative perspective for further research on the evaluation of high-temperature property indicators of SBS/CRM modified asphalt binders.

## 2. Materials and Methods

### 2.1. Materials

The based asphalt binders used in the test are Shell Pen #70 and Korea Shuang long Pen #70. The properties of the two asphalt binders are shown in Table 1. The modifiers used in the test were CRM and SBS. The size of the CRM was 40 mesh, which was provided by Suzhou Medium Glue Resource Recycling Company, and its properties are shown in Table 2. The SBS is thermoplastic styrene-butadiene rubber of Sinopec model YH-791H, and its properties are shown in Table 3.

**Figure 1:**
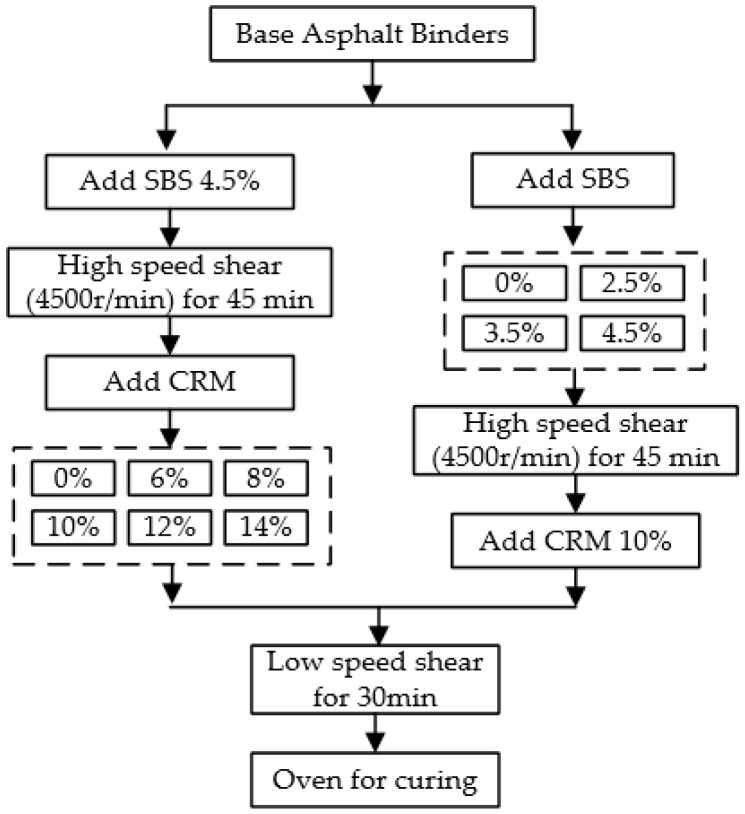
Production of hybrid modified binders with SBS and CRM.

For modified binders with a fixed SBS of 4.5 and various doses of CRM, the hybrid modified asphalt binders were made in the following way: Heat and melt a base asphalt binder to 135 °C, firstly; add 4.5% SBS in the melted binder and then mix the binder and the SBS with a high speed shear mixer at 4500 r/min for 45 min under 190 °C; then stir, separately, with CRM at a content of 0, 6, 8, 10, 12, and 14% for another 30 min with a low speed mixer under 190 °C; lastly, the hybrid asphalt binders were placed in an oven at 180 °C for 2 h for further curing. For the modified asphalt binders with a fixed CRM of 10% and various doses of SBS, repeat the process above; refer to the experiment in the public paper [19]. The flow chart of the hybrid modified asphalt binders produced can be seen in Figure 1.

### 2.2. Methods

#### 2.2.1. Three Indexes of Asphalt Binders

The three major indexes, i.e., penetration, softening point, and ductility of asphalt binders were tested in accordance with the Testing Procedures for Asphalt and Asphalt Mixtures for Highway Engineering of the Ministry of Transport of the People’s Republic of China in 2011 [3].

#### 2.2.2. Rheological Properties and Shear Viscosity by Dynamical Shear Rheometer (DSR)

A dynamic shear rheometer was used to measure the rheological and shear viscosity properties [20], instrument model: BOHLIN CVO 100D Dynamic Shearing Rheometer.

#### 2.2.3. ZSV at 60 °C and Its Calculation Model

The value of the ZSV at 60 °C is obtained through several steps. First, measure viscosity at different shear rates (0–100 HZ) by applying various torque and disk rotation speeds of the DSR test [14]. Then, plot the curves of the viscosity with a shear rate. Finally, to fit the curves using a rheology Carreau model [21], the Carreau model formula is shown in Equation (1).

(1) $$\frac{\eta -\eta_{\infty }}{\eta_{0}-\eta_{\infty }}=\frac{1}{{\left[1+{\left(\kappa \omega \right)}^{2}\right]}^{m/2}}$$

*η*—viscosity, Pa·s; *ω*—shear rate, 1/s; *k*, *m*—characteristic constant of material; *η*_0_—First Newtonian viscosity, i.e., ZSV, Pa·s; *η_∞_—*viscosity of the Second Newtonian region of the flow curve, Pa·s.

#### 2.2.4. Dynamic Viscosity at 60 °C

According to testing procedures [3], following the Hagen–Poiseu Law, measure the time taken for the asphalt binder to flow through the capillary section of the Newtonian fluid. The asphalt viscosity characterized the bonding properties and reflected the amount of internal inter-molecular frictional resistance when the asphalt binder flowed.

#### 2.2.5. Rotation Viscosity at 135 °C

Using a Brookfield viscometer, the Brookfield viscosity value of the pitch was obtained from the measured torque based on the viscosity of the asphalt itself.

#### 2.2.6. Gray Correlation Analysis Method

Gray correlation analysis method step [22]:

(1) Determine the original reference sequence and set a discrete sequence as follows:
  *X*_0_(*k*), *k* = 1, 2, 3, …, *m*
  *X_i_*(*k*), *k* = 1,2,3, …, *m*; *i* = 1,2,3, …, *n*
  *X*_0_(*k*) is the original reference sequence; *X_i_*(*k*) is the reference sequence.
(2) The data is dimensionless, that is, a mean processing method is adopted.
  (2) $$Y_{0}\left(k\right)=\frac{x_{0}(k)}{\sum_{k=1}^{m}X_{0}(K)/m},\;k=1,2,\;\ldots ,\;m$$
  (3) $$Y_{i}(k)=\frac{x_{i}(k)}{\sum_{k=1}^{m}X_{i}(k)/m},\;k=1,2,\;\ldots ,\;m;\;i=1,2,\;\ldots ,\;n\;$$
(3) Calculate the gray correlation coefficient. If the number obtained after the averaging process is {*X_0_*(*t*)} and the number of records is {*X_i_*(*t*)}, then at time t = k, the association between {*X*_0_(*t*)} and {*X*_i_(*t*)} the coefficient ξ0*i*(*k*) is calculated by the following formula.

(4) $$\xi 0i\left(k\right)=\left|\frac{\Delta_{\min}+\rho \Delta_{\max}}{\Delta_{0i}\left(k\right)+\rho \Delta_{\max}}\right|$$

Δ0*i*(*k*) is the absolute difference between two sequences at time k, that is, Δ0i(k) = |X0(k)-*X_i_*(*k*)|; Δmax is the maximum value of the absolute difference at each time; Δ_min_ is the minimum value of the absolute difference at each time; ρ is the resolution coefficient, which is usually 0.5;

(4) Calculate the degree of relevance. The formula for calculating the gray correlation degree is:

(5) $$r_{0i}=\frac{1}{m}\sum_{k=1}^{m}\xi_{0i}(k),\;k=1,2,3,\;\ldots ,\;m;\;i=1,2,3,\;\ldots ,\;n$$

*r*_0*i*_ is the degree of association between the curve *Y_i_* and *Y*_0_.

**Figure 2:**
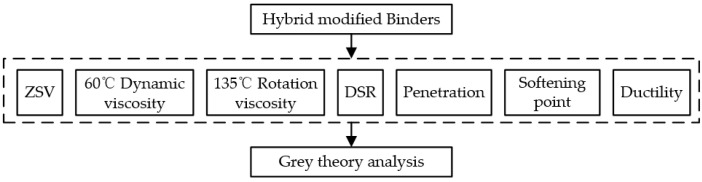
Test route of the properties of Hybrid modified binders.

The test flow chart is shown in Figure 2.

## 3. Results and Discussions

### 3.1. Shear Viscosity at Different Frequencies

**Figure 3:**
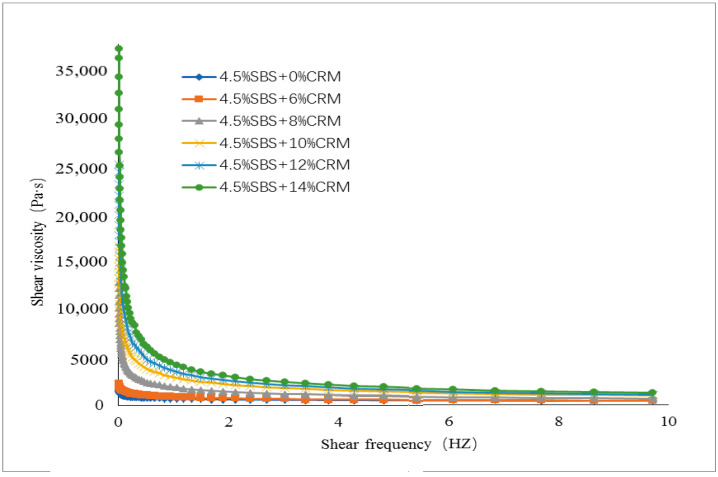
ZSV curves of Shuang long modified asphalt binder (fixed SBS and various CRM).

**Figure 4:**
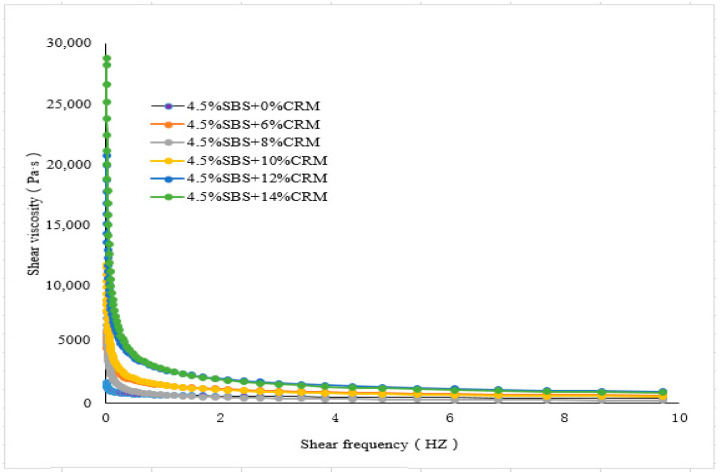
ZSV curves of Shell modified asphalt binder (fixed SBS and various CRM).

**Figure 5:**
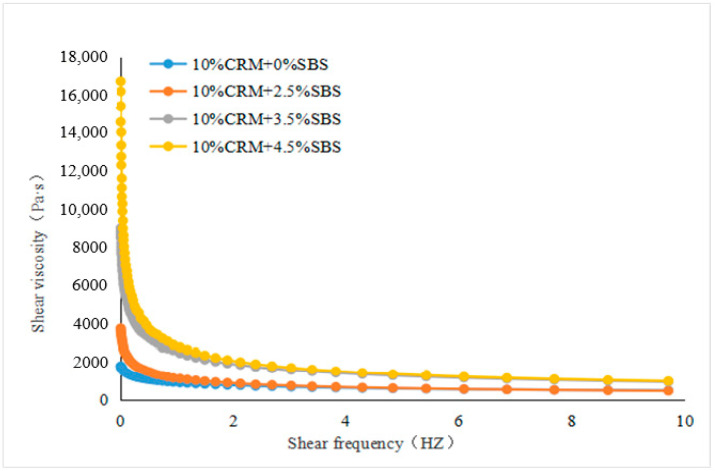
ZSV curves of Shuang long modified asphalt binder (fixed CRM and various SBS).

**Figure 6:**
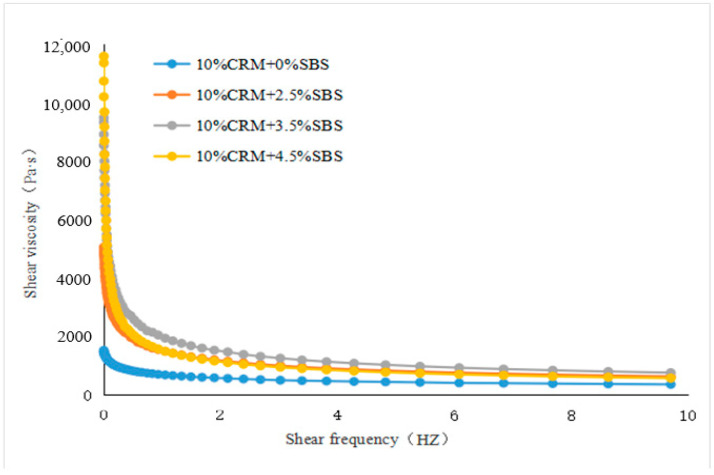
ZSV curves of Shell modified asphalt (fixed CRM and various SBS).

The values of shear viscosity at various frequencies were measured. ZSV was a value that was obtained when the shear frequency tends to zero from the curves fitted to the shear viscosity and shear frequency, see Figure 3, Figure 4, Figure 5 and Figure 6. To have a close look at the value of ZSV, the curves were fitted using those data collected with low shear frequencies up to 10 Hz. The frequency was selected by 64 points for measurement. The closer the frequency was to zero, the denser the points selected.

Figure 3, Figure 4, Figure 5 and Figure 6 showed the trends of ZSV of the hybrid modified binders with different shear rates at the test temperature of 60 °C. It can be seen that they have a similar trend: the value of ZSV decreased as the shear rate increased. A sharp decrease of ZSV was ob-served when the shear rate was less than 1 Hz, followed by a slow decrease. These findings are true for the two cases of base binders. The curves showed that the shear rate was a power function with the viscosity.

### 3.2. ZSV at 60 °C

Using the software Origin Pro 8 to fit the data, the values of ZSV at 60 °C of the modified asphalt binders were obtained. For values of ZSV of hybrid modified binders with a fixed SBS of 4.5%, see Table 4.

**Figure 7:**
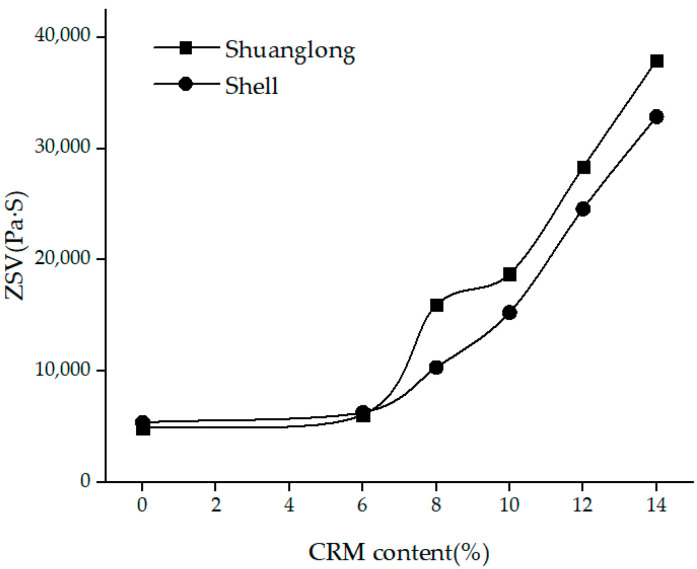
ZSV of hybrid modified asphalt binders with different CRM content.

It can be seen from Table 4 and Figure 7 that the values of the ZSV of the hybrid modified asphalt binders with a fixed SBS increased significantly with the increase of CRM content. The noticeable effectiveness of CRM on the hybrid modified asphalt binders was observed regardless of the type of the base asphalt binders. When adding 6% CRM, the value of the ZSV of hybrid modified asphalt with Shell base binder was 6.3 × 103 Pa·s and increased from 5.4 × 10^3^ Pa·s of 0% CRM binders, while the value of the ZSV with Shuang long base asphalt binder was 6.1 × 103 Pa·s and again increased, from 4.9 × 103 Pa·s of 0% CRM binders.

Continuous increases from 6%, 8%, 10%, 12%, and 14% CRM, made a linear increase in the ZSV. Overall, an increase of 8% from 6–14% of CRM in the hybrid binders made an increase rate of 420% and 520% for a base asphalt binder of Shell and Shuang long, respectively. For values of the ZSV of hybrid modified asphalt binders with a fixed 10% CRM, see Table 5.

**Figure 8:**
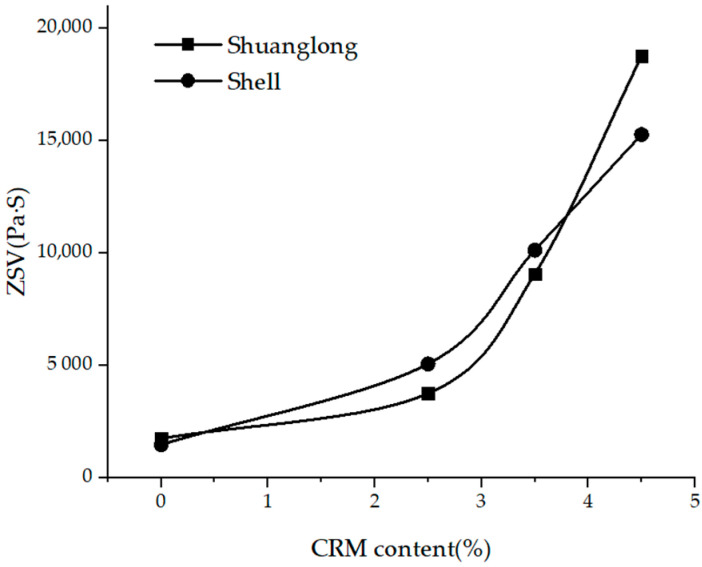
ZSV of hybrid modified asphalt binders with different SBS contents.

It can be seen from Table 5 and Figure 8 that the values of the ZSV of the hybrid asphalt binders with a fixed CRM of 10% increased significantly with the increase of SBS content also. The effectiveness of SBS on the hybrid modified binders was observed regardless of the type of the base binder. By adding 2.5% SBS, the value of the ZSV of the hybrid modified asphalt with Shell base binder was 5.08 × 103 and Pa·s increased from 1.49 × 10^3^ Pa·s of 0% SBS binders, while the value of the ZSV with Shuang long base binder, which was was 3.77 × 10^3^ Pa·s, again increased, from 1.76 × 10^3^ Pa·s of 0% SBS binders.

Continuous increases from 2.5%, 3.5% to 4.5% SBS increased the ZSV of the hybrid linearly. An addition of 2.0% CRM in the hybrid modified asphalt binders made a linear increase of 200% and 400%, respectively, for Shell and Shuang long base asphalt binder. These findings indicated that the SBS dose on the ZVS was really effective and the effectiveness of the increase also is dependent on the type of base asphalt binder.

In general, both the modifiers of SBS and CRM used for the hybrid modification were effective in producing high ZSV of hybrid modified asphalt binders, and the hybrid modified asphalt binders can achieve high values of ZSV by adding different combinations of the doses of SBS and CRM [23]. In other words, a part of SBS can be replaced by CRM in making hybrid modified asphalt binders and vice versa.

### 3.3. Gray Correlation between ZSV and Rheological Parameters

#### 3.3.1. ZSV and the Other Properties

The values of ZSV obtained by Carreau model fitting hybrid modified asphalt binders with different CRM contents are taken as the reference sequence X0, and the asphalt penetration, softening point, ductility at 5 °C, dynamic viscosity at 60 °C, Brinell rotation viscosity at 135 °C and composite shear modulus, phase angle, and rutting factor at 76 °C (X1, X2, X3, X4, X5, X6, X7, X8) are selected as comparison series. The factors of the reference sequence and the comparison sequence are summarized as shown in Table 6 and Table 7 below. The test data in the table is based on the performance test of the hybrid modified asphalt binders which is prepared by fixing 4.5% SBS and changed CRM contents.

**Figure 9:**
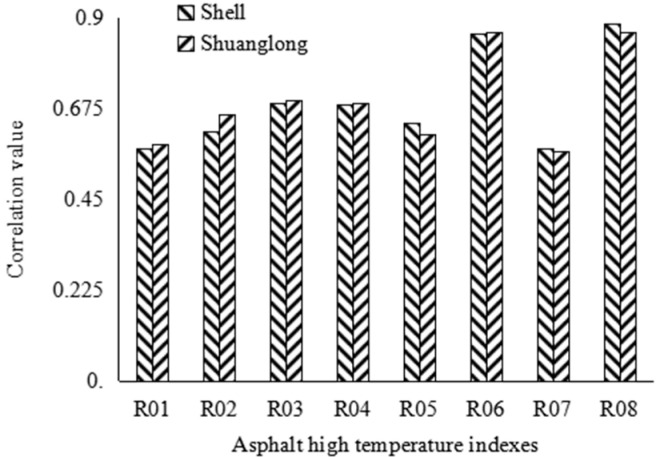
Gray correlation analysis results of ZSV and the other properties.

The data of Table 6 and Table 7 are calculated and processed by the formula of the gray correlation degree, and the correlation degree values of each factor can be obtained, as shown in Table 8 and Figure 9.

It can be seen from Figure 9 that the gray correlation analysis results between the ZSV and the other properties of asphalt binders is: *r*_08_ > *r*_06_ > *r*_03_ > *r*_04_ > *r*_02_ > *r*_05_ > *r*_01_ > *r*_07_ for Shuang long, and *r*_08_ > *r*_06_ > *r*_03_ > *r*_04_ > *r*_05_ > *r*_02_ > *r*_01_ > *r*_07_ for Shell base asphalt binder, respectively.

The results show the anti-rutting factor (G*/sinθ) has the highest correlation with asphalt ZSV, the composite shear modulus (G*) is the second, and the phase angle (θ) is the lowest related to ZSV. ZSV is an important index for the rutting of asphalt pavement, characterizing the viscoelastic properties of asphalt. It is reasonable to use ZSV to evaluate the high temperature performance of asphalt binder.

#### 3.3.2. Anti-Rutting Factor and Other Properties

**Figure 10:**
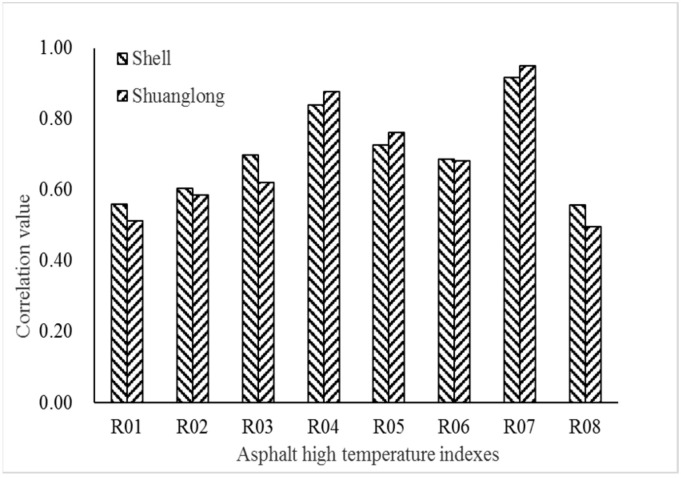
Gray correlation analysis results of anti-rutting and the other properties.

The anti-rutting factor is taken as a reference series, and the asphalt penetration, softening point, ductility at 5 °C, dynamic viscosity at 60 °C, Brinell rotation viscosity at 135 °C, ZSV, composite shear modulus and phase angle at 76 °C are selected as comparison series. Gray correlation calculation was conducted again. The results are shown in Table 9 and Figure 10.

It can be seen from Figure 10 that the results of the two types of base asphalt binders are similar. The highest correlation with the anti-rutting factor gray is the composite shear modulus, followed by the ZSV. The anti-rutting factor is calculated from the composite shear modulus, so the correlation degree is the highest. The second highest correlation with the ZSV gray indicates that the ZSV can well characterize the high temperature performance of asphalt binder, so it is reasonable to use ZSV as a key indicator for the evaluation of high viscosity modified asphalt binder.

## 4. Conclusions

This project was to examine ZSV of hybrid modified asphalt binders with crumb rubber modifier (CRM)/Styrene-butadiene-styrene (SBS) and the relationships of the ZSV and other properties of the modified asphalt binders based on grey theory. Two kinds of different base asphalt binders were used to prepare hybrid modified asphalt binders for the ZSV, dynamic viscosity at 60 °C, rotation viscosity at 135 °C, penetration, Ring and Ball (R&B) soft point, and ductility. The effects of modifier content of CRM/SBS on ZSV were investigated. The Carreau rheological model was used to correlate the relationships between the ZSV at 60 °C and the other properties. Conclusions can be made as followings:

(1) The high-viscosity of hybrid modified asphalt binders with SBS and CRM exhibits non-Newtonian fluid characteristics under high temperature conditions, and its flow curve can be fitted by the Carreau model to obtain the zero shear viscosity value (ZSV);
(2) The ZSV of hybrid modified asphalt binders increased significantly (at a fixed 4.5% SBS) with the addition of CRM at a content of 6–14%. The ZSV of hybrid modified binders also increased significantly (at a fixed 10% CRM) with the increase of SBS content from 0–4.5%;
(3) The modifiers of both SBS and CRM are effective to increase the ZSV of the hybrid modified binders. High viscosity of hybrid modified binders can be achieved by either addition of SBS or CRM;
(4) Gray correlation analysis showed that the ZSV of asphalt binders has the highest correlation with anti-rutting factor (G*/sinθ), which indicated that ZSV is another good indicator of the viscoelastic properties of high-viscosity modified asphalt binders.

## Figures and Tables

**Table 1:** Properties of base asphalt binders.

Properties	Shell	Shuang Long	Standard
Penetration at 25 °C/(0.1 mm)	71	72	60~80
Ductility at 15 °C (5 cm/min)	106	108	≥100
Softening point/°C	48	48.5	≥46
Dynamic viscosity at 60 °C/(Pa·s)	224	180	≥180

**Table 2:** Properties of 40 mesh CRM.

Properties	Actual Value	Standard
Moisture/%	0.80	0~2.0
Apparent density/(g/cm^3^)	0.36	0.27~0.39
Ash/%	7.4	0~8.5

**Table 3:** Properties of SBS (YH-791H).

Properties	Actual Value
Volatile matter/%	1.0
300% Tensile stress/MPa	2.0
Ash/%	0.2
Elongation at break/%	700
Melt flow rate/(g/10 min)	0.01–0.50
Hardness	68

**Table 4:** ZSV Values of hybrid modified asphalt binders (fixed SBS and various CRM).

Asphalt Binders	Shell/(Pa·s)	Shuang Long/(Pa·s)
4.5%SBS + 0%CRM	5.4 × 10^3^	4.9 × 10^3^
4.5%SBS + 6%CRM	6.3 × 10^3^	6.1 × 10^3^
4.5%SBS + 8%CRM	10.33 × 10^3^	15.95 × 10^3^
4.5%SBS + 10%CRM	15.28 × 10^3^	18.76 × 10^3^
4.5%SBS + 12%CRM	24.59 × 10^3^	28.33 × 10^3^
4.5%SBS + 14%CRM	32.88 × 10^3^	37.93 × 10^3^

**Table 5:** ZSV values of hybrid modified asphalt binders (fixed CRM and various SBS).

Asphalt Binders	Shell/(Pa·s)	Shuang Long/(Pa·s)
0%SBS + 10%CRM	1.49 × 10^3^	1.76 × 10^3^
2.5%SBS + 10%CRM	5.08 × 10^3^	3.77 × 10^3^
3.5%SBS + 10%CRM	10.14 × 10^3^	9.08 × 10^3^
4.5%SBS + 10%CRM	15.28 × 10^3^	18.76 × 10^3^

**Table 6:** Gray correlation analysis of ZSV and the other properties for the hybrid modified asphalt binders with Shuang long base binder.

CRM Content/%		0	6	8	10	12	14
ZSV/(KPa·s)	X0	4.94	6.11	15.95	18.76	28.33	37.93
Penetration/(0.1 mm)	X1	57.8	48	44.4	41.2	36.84	38.75
Softening point/°C	X2	78.4	80.5	82.8	88.7	91.4	92.6
5 °C Ductility/cm	X3	22.5	12.1	22.8	21.4	20.2	34.7
60 °C Viscosity/(Pa·s·10^3^)	X4	9.69	16.75	38.02	106.07	255.84	322.55
135 °C Viscosity/(Pa·s·10^3^)	X5	1.04	3.89	4.50	8.20	42.30	56.20
Composite Shear Modulus/KPa	X6	1.08	2.07	4.05	6.58	10.21	10.72
Phase angle/θ	X7	78.25	70.36	58.02	52.31	46.83	45.46
Anti-rutting factor/KPa	X8	1.10	2.19	4.78	8.32	14.00	15.04

**Table 7:** Gray correlation analysis of ZSV and the other properties for the hybrid modified asphalt binders with Shell base binder.

CRM Content/%		0	6	8	10	12	14
ZSV/(KPa·s)	X0	5.42	6.34	10.33	15.28	24.59	32.88
Penetration/(0.1 mm)	X1	54.57	49.67	48.2	43.7	39	39.1
Softening point/°C	X2	74.4	75.9	78.8	86.7	88.9	88.8
5 °C Ductility/cm	X3	24.3	11.5	19.4	23.8	24.1	32.6
60 °C Viscosity/(Pa·s·10^3^)	X4	10.64	11.65	37.15	84.55	215.21	275.16
135 °C Viscosity/(Pa·s·10^3^)	X5	1.31	4.27	4.31	7.10	31.10	52.30
Composite Shear Modulus/KPa	X6	1.43	3.31	4.08	4.75	8.95	9.15
Phase angle/θ	X7	75.26	66.19	56.94	53.41	45.62	46.55
Anti-rutting factor/KPa	X8	1.47	3.62	4.87	5.91	12.52	12.61

**Table 8:** Correlation degree of ZSV and the other properties.

Relevant Influencing Factors	Correlation Degree	Shuang Long	Shell
Penetration/(0.1 mm)	r_01_	0.587	0.576
Softening point/°C	r_02_	0.660	0.617
5 °C Ductility/cm	r_03_	0.697	0.688
60 °C Viscosity/(Pa·s·10^3^)	r_04_	0.688	0.685
135 °C Viscosity/(Pa·s·10^3^)	r_05_	0.612	0.640
Composite Shear Modulus/KPa	r_06_	0.864	0.862
Phase angle/θ	r_07_	0.569	0.575
Anti-rutting factor/KPa	r_08_	0.866	0.884

**Table 9:** Correlation degree of anti-rutting and the other properties.

Relevant Influencing Factors	Correlation Degree	Shuang Long	Shell
Penetration/(0.1 mm)	*r*_01_	0.514	0.559
Softening point/°C	*r*_02_	0.585	0.605
5 °C Ductility/cm	*r*_03_	0.622	0.698
60 °C Viscosity/(Pa·s·10^3^)	*r*_04_	0.762	0.726
135 °C Viscosity/(Pa·s·10^3^)	*r*_05_	0.682	0.687
ZSV/(KPa·s)	*r*_06_	0.876	0.839
Composite Shear Modulus/KPa	*r*_07_	0.951	0.917
Phase angle/θ	*r*_08_	0.497	0.558
